# Comparison of two maxillary protraction protocols: tooth-borne versus bone-anchored protraction facemask treatment

**DOI:** 10.1186/s40510-015-0096-7

**Published:** 2015-08-25

**Authors:** Peter Ngan, Benedict Wilmes, Dieter Drescher, Chris Martin, Bryan Weaver, Erdogan Gunel

**Affiliations:** Department of Orthodontics, School of Dentistry, West Virginia University, 1073 Health Science Center North, P.O. Box 9480, Morgantown, WV 26506 USA; Department of Orthodontics, University of Düsseldorf, Moorenstrasse, Germany; Department of Oral and Maxillofacial Surgery, School of Dentistry, West Virginia University, Morgantown, USA; Department of Statistics, West Virginia University, Morgantown, USA

## Abstract

**Background:**

Protraction facemask has been advocated for treatment of class III malocclusion with maxillary deficiency. Studies using tooth-borne rapid palatal expansion (RPE) appliance as anchorage have experienced side effects such as forward movement of the maxillary molars, excessive proclination of the maxillary incisors, and an increase in lower face height. A new Hybrid Hyrax bone-anchored RPE appliance claimed to minimize the side effects of maxillary expansion and protraction. A retrospective study was conducted to compare the skeletal and dentoalveolar changes in patients treated with these two protocols.

**Methods:**

Twenty class III patients (8 males, 12 females, mean age 9.8 ± 1.6 years) who were treated consecutively with the tooth-borne maxillary RPE and protraction device were compared with 20 class III patients (8 males, 12 females, mean age 9.6 ± 1.2 years) who were treated consecutively with the bone-anchored maxillary RPE and protraction appliances. Lateral cephalograms were taken at the start of treatment and at the end of maxillary protraction. A control group of class III patients with no treatment was included to subtract changes due to growth to obtain the true appliance effect. A custom cephalometric analysis based on measurements described by Bjork and Pancherz, McNamara, Tweed, and Steiner analyses was used to determine skeletal and dental changes. Data were analyzed using a one-way analysis of variance.

**Results:**

Significant differences between the two groups were found in 8 out of 29 cephalometric variables (*p* < .05). Subjects in the tooth-borne facemask group had more proclination of maxillary incisors (OLp-Is, Is-SNL), increase in overjet correction, and correction in molar relationship. Subjects in the bone-anchored facemask group had less downward movement of the “A” point, less opening of the mandibular plane (SNL-ML and FH-ML), and more vertical eruption of the maxillary incisors.

**Conclusions:**

The Hybrid Hyrax bone-anchored RPE appliance minimized the side effect encounter by tooth-borne RPE appliance for maxillary expansion and protraction and may serve as an alternative treatment appliance for correcting class III patients with a hyperdivergent growth pattern.

## Background

Several studies have recommended early treatment of developing class III malocclusion for growth modification [1–5]. The validity of two-phase treatment is supported by studies that show greater orthopedic response when treatment is started in younger patients [6, 7]. Other studies reported early correction of the malocclusion allows for a favorable growth environment for dentofacial development and may help in preventing development into more severe malocclusion in late adolescence [8]. Early class III treatment using protraction facemask in conjunction with rapid palatal expansion (RPE) appliance has been shown to be successful in correcting skeletal class III malocclusions that are due primarily to deficient maxillary development [9–11]. The protraction facemask generates an anteriorly directed force on the maxilla. The forces act indirectly on the circummaxillary sutures, which are still patent at an early age, and thereby stimulate bone apposition in the suture areas. The goal of combining the RPE with protraction facemask is to provide a more effective protraction of the maxilla by disarticulating the circummaxillary sutures [12, 13].

Conventional protraction facemask therapy, with an indirect application of force to the sutures through tooth-borne anchorage, causes both skeletal and dental changes because the applied force was directed below the center of resistance of the maxilla, resulting in a counterclockwise rotation of the maxilla, backward rotation of the mandible, labial tipping of the maxillary incisors, and lingual tipping of the mandibular incisors. This is usually accompanied by an increase in lower face height and a decrease in overbite [9–12]. The goal of protraction facemask therapy is to obtain pure skeletal changes with minimal undesirable dental effects. Previous studies have shown that tooth-borne protraction facemask therapy have undesirable side effects such as excessive forward movement and extrusion of the maxillary molars, excessive proclination of the maxillary incisors, and an increase in lower face height [14–17]. This is a concern especially in situations in which preservation of arch length is necessary. Attempt has been made in designing an absolute anchorage system for maxillary protraction. These newer treatment modalities include the use of intentionally ankylosed maxillary deciduous canines [18], osseointegrated titanium implants [19, 20], onplants [21], miniscrews, and most recently miniplates [22–33]. Each implant system has strengths and weaknesses. The use of osseointegrated titanium, onplants, and miniplates required invasive surgical procedures. To simplify the placement of bone-anchored devices, Wilmes et al. introduced the Hybrid Hyrax RPE appliance, a tooth- and bone-anchored device [23, 24, 32, 33]. Two mini-implants are placed in the anterior palate, and an expansion device is connected to the mini-implants and the first molars. After expansion, the patient undergoes facemask therapy with elastics attached to the hooks on the Hybrid Hyrax expansion appliance. Several case reports have been published on the application of this device [23, 24, 32, 33]. To date, no literature has been presented comparing the results of the Hybrid Hyrax bone-anchored protraction facemask with the tooth-borne protraction facemask treatment. The purpose of this study is to compare the skeletal and dentoalveolar changes between the two treatment protocols. The null hypothesis is that there is no difference in treatment effects for the sagittal, vertical, and angular changes between the two maxillary protraction protocols.

## Methods

This study was approved by the Institutional Review Board of West Virginia University (1401168542). Approval was also granted from one of the authors (B.W.) for the use of orthodontic records from his office. The study groups consisted of 20 patients (8 males, 12 females) who were treated consecutively by the tooth-borne RPE appliance and protraction facemask and 20 patients (8 males, 12 females) who were treated consecutively by the bone-anchored Hybrid Hyrax expansion appliance and protraction facemask. Table 1 shows the age distribution of the two experimental groups. The inclusion criteria for patient selection were class III malocclusion at the time of the initial observation (T1) as defined by one or more of the following characteristics: anterior crossbite or edge-to-edge incisal relationship; accentuated mesial step or class III permanent molar relationship; a Wits appraisal smaller than −3 mm or an ANB smaller than −2°. The exclusion criteria were no prior orthopedic or orthodontic treatment and no craniofacial syndromes. A sample consisted of 20 class III patients with no treatment who were under observation for class III treatment and matched closely in age and craniofacial morphology with the study groups were used as the control group.

**Table 1:** Age distribution for the control, tooth-borne, and bone-anchored protraction facemask groups

	Control			Tooth-borne protraction facemask			Bone-anchored protraction facemask		
	t0	t1	Diff	t1	t2	Diff	T1	T2	Diff
Mean	9.0	9.8	0.8	9.7	10.5	0.8	9.8	10.4	0.6
SD	1.8	1.6	1.6	1.6	1.6	1.6	1.2	1.2	1.2
Min	6.1	6.9		6.90	7.11		8.0	9.0	
Max	12.7	13.2		13.2	14.1		12.0	13.0	

### Appliances for class III correction

**Fig. 1:**
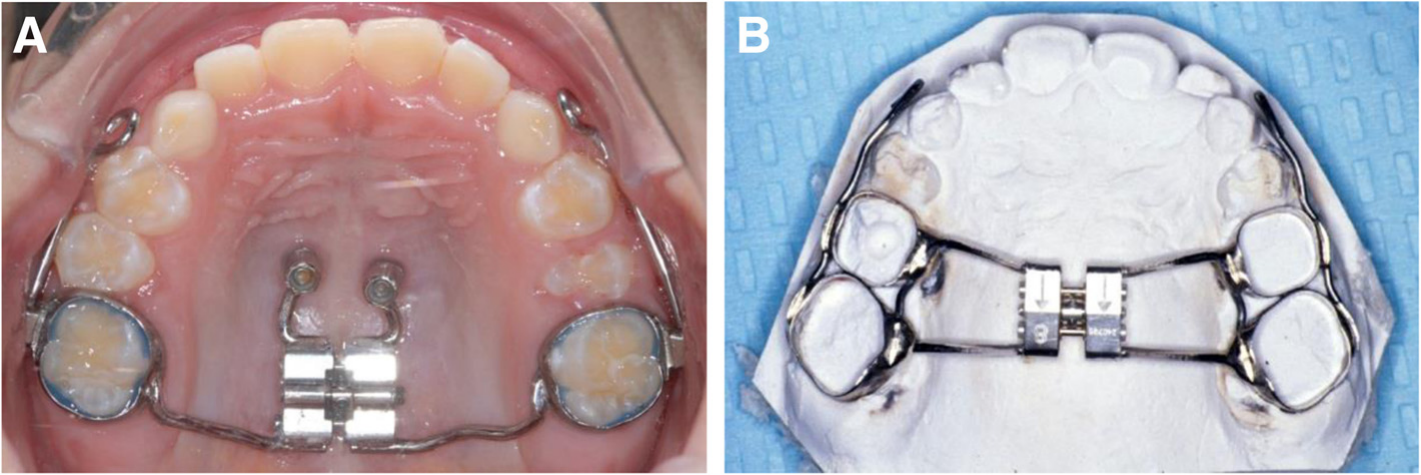
**a** Bone-anchored Hybrid Hyrax expansion appliance. **b** Tooth-borne Hyrax expansion appliance

The tooth-borne RPE was constructed by placing bands on the posterior teeth (Fig. 1a). Bands were fitted on the maxillary primary second molars and permanent first molars. These bands were joined by a heavy wire (.043 in.) to the palatal plate, which had a jack screw in the midline. The appliance was activated twice daily (0.25 mm per turn) by the patient for 1 week. In patients with a constricted maxilla, activation of the expansion screw was applied for 2 weeks. A 0.045-in. wire was soldered bilaterally to the buccal aspects of the molar bands and extended anteriorly to the canine area with a hook for protraction elastics.

The bone-borne Hybrid Hyrax RPE was constructed by placing bands on the permanent first molars (Fig. 1b). Two Benefit micro-implants (2 mm × 9 mm, PSM Medical Solutions, Tuttlingen, Germany) were placed in the anterior palate, in the area of the third palatal rugae. Transfer caps were added before a silicone impression was taken. Laboratory analogs were placed over the transfer caps, bands were positioned in the impression, and a plaster cast is made. Two standard Benefit abutments were screwed over the laboratory analogs. A Hyrax-type (Trademark of Dentaurum, Inc., Newtown, PA) palatal split screw was welded or soldered to the two anterior abutments and to the molar bands. The jackscrew was activated twice daily (0.25 mm per turn) by the patient for 1 week. In patients with a constricted maxilla, activation of the expansion screw was applied for 2 weeks. Rigid .048-in. stainless steel sectional wires with hooks near the canines are welded or soldered to the buccal sides of the molar bands for the application of orthopedic protraction forces.

The facemask was a one-piece construction with an adjustable anterior wire and hooks to accommodate a downward and forward pull of the maxilla with elastics. The protraction elastics were attached near the maxillary canines with a downward and forward pull of 30° to the occlusal plane. Maxillary protraction generally requires an orthopedic force of 300–600 g per side depending on the age of the patient. In the present study, elastics that delivered 380 g per side as measured by a force gauge were used. The patients were instructed to wear the facemask for 12–14 h a day.

### Cephalometric analysis

**Fig. 2:**
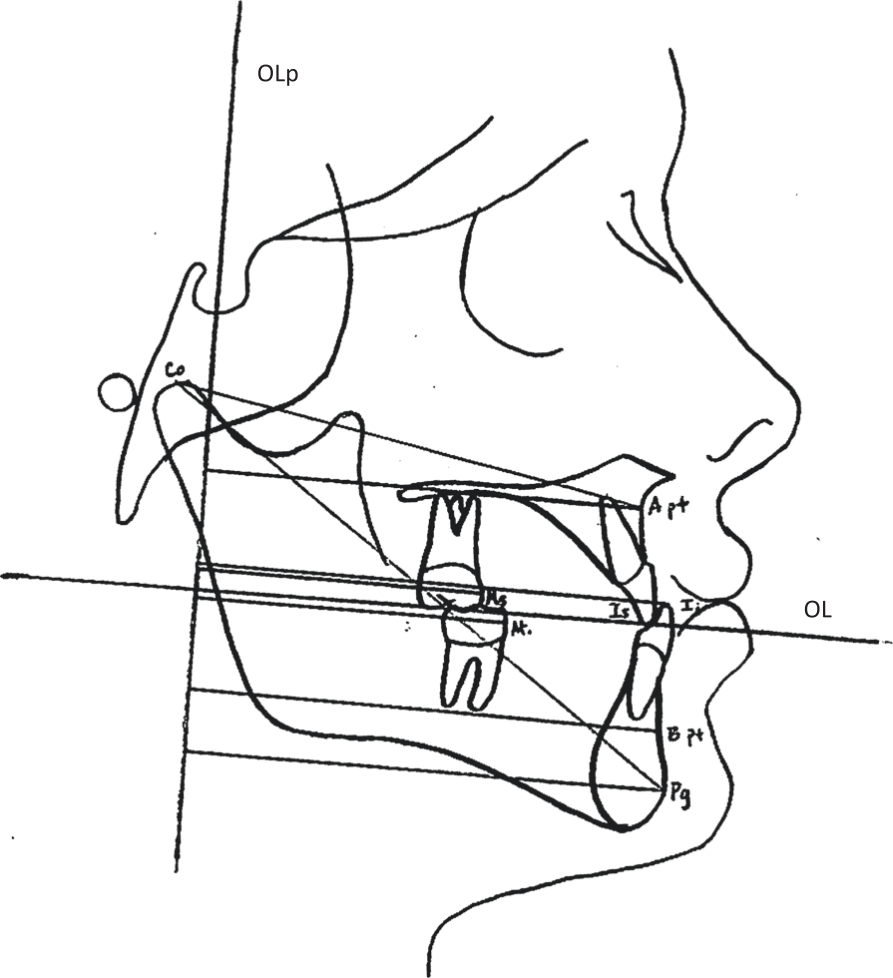
A reference grid was constructed using occlusal plane (*OL*) and occlusal plane perpendicular (*OLp*). All sagittal measurements were made from the reference grid to the landmarks A point: upper incisal tip (*Is*); lower incisal tip (*Ii*); *B* point; pogonion (*Pg*); mesial buccal cusp of upper molar (*Ms*); and mesial buccal cusp of lower molar (*Mi*)

**Fig. 3:**
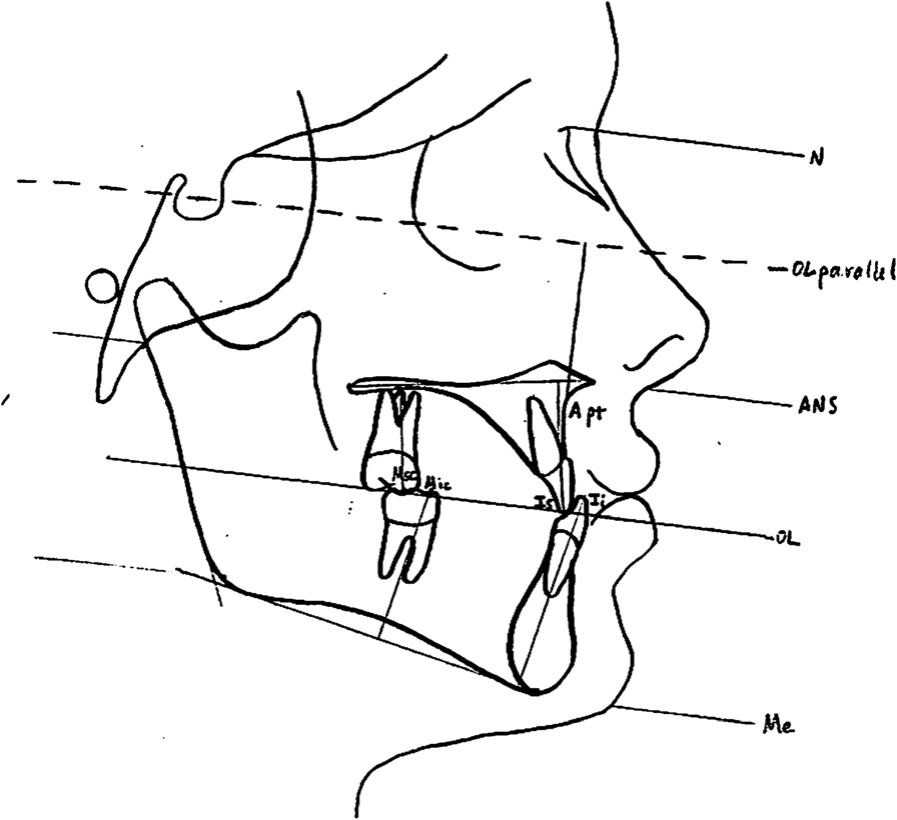
Landmarks used in vertical measurements. Vertical changes of *A* point was measured from the occlusal plane parallel (*OLp*); maxillary incisal and molar changes were measured from the palatal plane; mandibular incisor and molar changes were measured from the mandibular plane

**Fig. 4:**
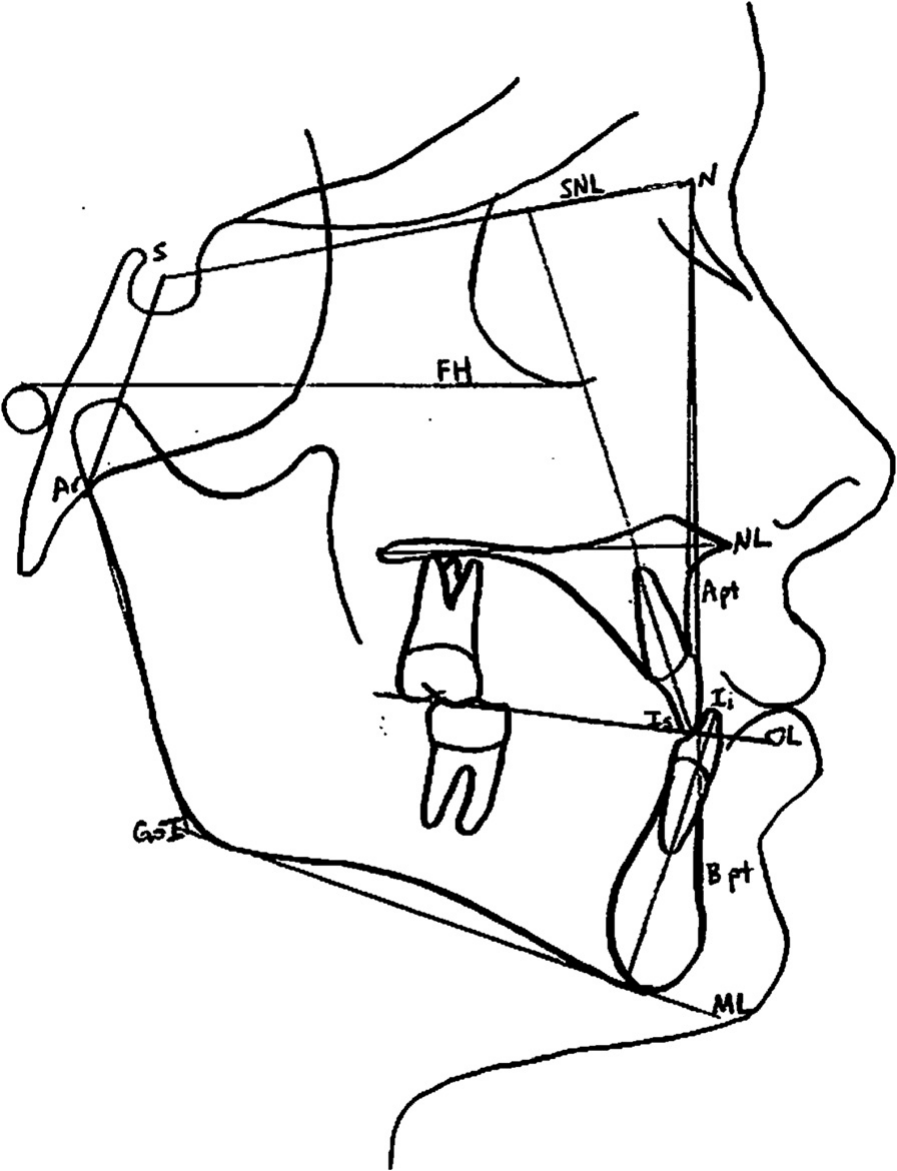
Landmarks used in angular measurements. Palatal plane, occlusal plane, and mandibular plane were measured with reference to sella nasion plane (*SNL*)

Pre- and post-treatment lateral cephalograms were digitized and calibrated using the Dolphin Imaging software (Dolphin Imaging, Chatsworth, CA, USA). A customized cephalometric system incorporating measurements from Bjork [35] and Pancherz [36] for the sagittal and vertical variables and Steiner, Tweed, and McNamara analyses for the angular variables were used to determine treatment changes. The landmarks used are shown in Figs. 2, 3, and 4. The magnification factor of the lateral cephalograms was found to be 6 % for both the control and the tooth-borne treatment groups. There was no magnification factor for the digital radiographs of the bone-borne treatment group. The sagittal and vertical variables were reported in millimeters and were recorded to the nearest 0.1 mm. Angular measurements were reported to the nearest 0.1°. Films were printed 1:1 using a Kodak ESP 7250 printer (Kodak, Atlanta, GA, USA) and then traced by one investigator using a 0.5 mm mechanical lead pencil, 3 M Unitek orthodontic protractor (Monrovia, CA), and 0.003 in. matte 3 M Unitek cephalometric acetate tracing film (Monrovia, CA). All radiographs were traced by one person (N.D.) who was blinded to the treatment groups to avoid bias to cephalometric tracing. Analysis of the sagittal skeletal and dental changes were recorded along the occlusal plane (OL) and to the occlusal plane perpendicular (OLp) from the first film, this formed the reference grid. The grid was then transferred to a subsequent film by superimposing the tracing on the mid-sagittal cranial structures. The changes in overjet and molar relationship were calculated using the formula depicted in (Table 2) using the formula depicted in Table 3.

**Table 2:** Calculation of changes in overjet and molar relationship

Overjet	Molar relationship
Skeletal contribution	Skeletal contribution
1. OLp–A pt.	1. OLp–A pt.
2. OLp–Pg	2. OLp–Pg
Dental contribution	Dental contribution
3. OLp–Is minus OLp–A pt.	3. OLp–Ms minus OLp–A pt.
4. OLp–Ii minus OLp–Pg	4. OLp–Mi minus OLp–Pg
Overjet correction	Molar relationship correction
Sum of 1, 2, 3, and 4	Sum of 1, 2, 3, and 4

**Table 3:** Sagittal, vertical, and angular measurements in the control group

Variables (mm)	T0				T1				T1 − T0
*Sagittal*	Mean	SD	Min	Max	Mean	SD	Min	Max	Diff
OLp–A pt.	67.27	3.73	60.19	74.21	68.06	3.90	60.58	75.17	0.80
OLp–B pt.	75.74	4.86	68.45	82.66	76.74	4.96	69.02	83.23	1.00
OLp–Pg	77.11	5.47	69.12	85.54	78.18	5.53	69.60	87.26	1.08
Wits	−8.52	3.10	−13.44	−2.50	−8.26	3.11	−13.63	−2.88	0.27
OLp–Is	74.17	4.68	62.40	80.74	75.49	4.92	63.55	83.90	1.32
OLp–Ii	76.26	4.78	63.94	82.75	77.48	4.95	65.86	85.63	1.22
Overjet	−2.09	0.98	−4.03	0.38	−1.99	0.90	−4.22	0.00	0.11
OLp–Ms	48.05	4.35	40.13	54.82	49.30	4.49	40.99	55.97	1.25
OLp–Mi	51.58	5.68	42.72	60.58	52.80	5.79	42.72	62.21	1.22
Molar relationship	−3.57	2.39	−7.97	0.48	−3.47	2.20	−7.87	−0.58	0.11
*Vertical (mm)*									
OLparallel–A pt.	20.35	38.78	29.59	5.89	23.81	40.42	31.47	5.62	1.88
Is–NL	20.26	29.86	24.91	2.69	20.64	30.62	25.74	2.61	0.83
Ii–ML	33.60	41.18	37.61	2.14	34.27	43.01	38.28	2.45	0.66
Overbite	0.00	10.46	3.01	2.56	0.00	9.02	3.32	2.36	0.31
Msc–NL	13.25	22.46	18.97	2.05	16.32	22.75	19.44	1.54	0.47
Mic–ML	25.06	30.91	28.32	1.46	26.30	30.62	28.62	1.25	0.30
*Angular (°)*									
SNA	73.63	81.60	78.57	2.59	73.82	82.75	78.85	2.85	0.29
SNB	73.82	83.04	78.75	2.92	74.02	83.42	79.27	2.76	0.52
ANB	−3.74	3.55	−0.18	2.09	−3.65	3.07	−0.41	2.02	−0.23
SNL–ML	24.00	37.44	32.58	4.54	23.42	38.21	32.09	4.59	−0.49
SNL–OL	12.48	28.80	20.17	5.40	12.58	27.94	19.96	4.99	−0.21
SNL–NL	3.36	12.58	8.62	3.02	1.73	12.38	7.88	3.21	−0.74
Is–SNL	85.73	117.31	100.78	8.62	87.36	116.54	103.17	8.31	2.39
Ii–ML	64.99	100.90	87.30	8.28	68.93	102.72	86.75	9.18	−0.56

### Data analysis

The statistical analysis was carried out utilizing the JMP version 90.9 SAS Software (Cary, NC). The starting forms of the control and experimental samples were compared using a two-tailed *t* test. The skeletal and dental changes between the treated and control sample at the two time periods were compared with a two-tailed *t* test. The confidence level was set at 95 %.

### Method error

The error in locating, superimposing, and measuring the changes of the landmarks by one examiner was measured on the cephalograms of 10 randomly selected subjects. All cephalograms were recorded twice independently on two separate occasions with a 2-week interval. For all the cephalometric variables, difference between the independent repeated measurements of each individual before/after treatment was recorded. The intraclass correlation coefficient of reliability (*R*) was used to determine the reliability of cephalometric measurements. The *R* value can range from 0 to 1.00 with *R* value greater than 0.90 indicating high reliability. The correlations of all the cephalometric variables ranged from 0.96 to 0.99, with most being above 0.98. The method of cephalometric analysis used in this study was deemed reliable and repeatable.

## Results

### Cephalometric changes

Changes in cephalometric measurements in the control group and patients treated with the tooth-borne and bone-borne protraction facemask are shown in Tables 3, 4, and 5. The appliance effect was calculated by subtracting the changes due to growth (control group) from the treatment changes (Table 6).

**Table 4:** Sagittal, vertical, and angular measurements for the tooth-borne protraction facemask group

Variables (mm)	T1				T2				T2 – T1
*Sagittal*	Min	Max	Mean	SD	Min	Max	Mean	SD	Diff
OLp–A pt.	60.58	75.17	68.06	3.90	60.77	75.94	69.59	4.26	1.53
OLp–B pt.	69.02	83.23	76.74	4.96	66.62	82.46	75.41	4.86	−1.33
OLp–Pg	69.60	87.26	78.18	5.53	66.34	85.15	77.14	5.58	−1.05
Wits	−13.63	−2.88	−8.26	3.11	−9.12	1.63	−5.74	3.23	2.52
OLp–Is	63.55	83.90	75.49	4.92	69.98	87.26	78.99	4.46	3.49
OLp–Ii	65.86	85.63	77.48	4.95	66.72	80.83	75.22	4.29	−2.27
Overjet	−4.22	0.00	−1.99	0.90	0.00	8.74	3.77	2.17	5.76
OLp–Ms	40.99	55.97	49.30	4.49	44.74	61.54	51.94	4.86	2.64
OLp–Mi	42.72	62.21	52.80	5.79	43.68	60.67	52.70	5.11	−0.12
Molar relationship	−7.87	−0.58	−3.47	2.20	−5.66	1.73	−0.75	2.06	2.72
*Vertical (mm)*									
OLparallel–A pt.	23.81	40.42	31.47	5.62	26.11	45.98	34.54	5.24	3.07
Is–NL	20.64	30.62	25.74	2.61	21.02	30.34	26.03	2.52	0.29
Ii–ML	34.27	43.01	38.28	2.45	35.42	42.82	39.02	1.91	0.75
Overbite	0.00	9.02	3.32	2.36	0.00	3.84	2.08	1.07	−1.24
Msc–NL	16.32	22.75	19.44	1.54	17.38	23.33	20.74	1.96	1.30
Mic–ML	26.30	30.62	28.62	1.25	27.07	31.87	29.60	1.47	0.98
*Angular (°)*									
SNA	73.82	82.75	78.85	2.85	75.36	84.19	79.55	2.78	0.69
SNB	74.02	83.42	79.27	2.76	72.77	81.41	77.54	2.28	−1.73
ANB	−3.65	3.07	−0.41	2.02	−2.59	6.34	2.01	2.54	2.42
SNL–ML	23.42	38.21	32.09	4.59	27.36	41.38	34.43	4.17	2.33
SNL–OL	12.58	27.94	19.96	4.99	11.04	24.10	17.98	4.20	−1.98
SNL–NL	1.73	12.38	7.88	3.21	4.51	12.00	7.89	2.04	0.01
Is–SNL	87.36	116.54	103.17	8.31	94.94	113.47	105.36	6.44	2.19
Ii–ML	68.93	102.72	86.75	9.18	72.96	96.00	81.75	7.09	−4.99

**Table 5:** Sagittal, vertical, and angular measurements for the bone-anchored protraction facemask group

Variables (mm)	T1				T2				T2 – T1
*Sagittal*	Min	Max	Mean	SD	Min	Max	Mean	SD	Diff
OLp–A pt.	60.0	76.9	66.74	3.93	61.9	78.7	68.28	4.32	1.54
OLp–B pt.	63.5	80.0	71.86	5.13	62.8	80.6	70.57	5.41	−1.29
OLp–Pg	66.7	84.0	73.95	4.70	65.8	84.1	73.82	5.11	−0.13
Wits	−9.6	0.2	−5.12	2.58	−6.9	6.9	−2.54	3.22	2.58
OLp–Is	60.1	79.3	70.28	4.67	60.3	84.4	72.47	5.57	2.19
OLp–Ii	61.9	79.0	71.14	4.67	55.4	80.9	69.88	5.52	−1.26
Overjet	−4.8	2.9	−0.86	2.31	−1.6	5.9	2.6	1.76	3.46
OLp–Ms	40.2	50.7	44.45	3.18	41.1	58.0	46.89	4.27	2.44
OLp–Mi	41.3	55.2	47.52	4.20	42.2	59.1	48.94	4.65	1.42
Molar relationship	−0.6	−7.0	−3.34	1.65	−4.9	0.4	−2.05	1.62	1.29
*Vertical (mm)*									
OLparallel–A pt.	16.4	38.9	30.14	5.43	16.5	41.3	31.62	6.07	1.48
Is–NL	14.4	27.7	22.41	3.69	19.9	29.0	24.58	2.61	2.17
Ii–ML	27.9	39.9	34.82	3.45	29.7	41.2	36.08	3.11	1.26
Overbite	−1.1	5.9	1.56	2.18	−1.8	4.4	1.42	1.48	−0.14
Msc–NL	9.1	21.9	17.02	3.00	11.8	22.9	18.58	2.73	1.56
Mic–ML	23.3	30.5	26.51	2.06	23.9	32.9	27.7	2.13	1.19
*Angular (°)*									
SNA	71.9	92.1	80.29	4.74	73.0	91.2	81.88	4.49	1.59
SNB	74.8	90.2	81.2	4.17	71.9	88.8	80.4	4.25	−0.8
ANB	−5.5	3.3	−0.92	2.78	−3.8	6.9	1.48	2.76	2.4
SNL–ML	22.2	44.9	32.62	6.07	21.1	49.5	32.86	7.13	0.24
SNL–OLs	9.0	29.4	18.14	5.89	7.2	32.0	17.31	6.09	−0.83
SNL–NL	2.6	12.9	6.44	3.07	1.1	14.0	6.52	3.68	0.08
Is–SNL	87.2	121.8	103.19	8.23	86.8	114.2	101.16	8.12	−2.03
Ii–ML	78.1	91.9	84.75	3.48	77.8	95.6	83.08	4.54	−1.67

**Table 6:** Comparison of differences between the tooth-borne and bone-anchored groups after subtracting changes due to growth (control group)

Variables	Tooth-borne protraction facemask		Bone-anchored protraction facemask			
*Sagittal*	Mean	SD	Mean	SD	*p* value	Sig
OLp–A pt.	0.72	1.29	0.74	1.23	0.98	NS
OLp–B pt.	−2.28	1.43	−2.31	2.15	0.93	NS
OLp–Pg	−2.07	2.11	−1.21	3.05	0.32	NS
Wits	2.19	2.74	2.31	2.40	0.89	NS
OLp–Is	2.12	1.22	0.87	2.43	0.03	*
OLp–Ii	−3.40	2.33	−2.48	2.26	0.23	NS
Overjet	5.53	2.00	3.35	2.45	0.003	*
OLp–Ms	1.35	1.53	1.19	1.78	0.67	NS
OLp–Mi	−1.31	0.55	0.20	2.93	0.06	NS
Molar relationship	2.53	1.55	1.18	1.99	0.02	*
*Vertical (mm)*						
OLparallel–A pt	1.15	2.00	−0.40	1.40	0.004	*
Is–NL	−0.52	1.50	1.34	2.80	0.01	*
Ii–ML	0.09	2.18	0.60	1.72	0.49	NS
Overbite	−1.49	2.90	−0.45	2.23	0.21	NS
Msc–NL	0.80	1.56	1.09	1.86	0.63	NS
Mic–ML	0.66	1.36	0.89	1.38	0.62	NS
*Angular (°)*						
SNA	0.39	1.74	1.29	2.13	0.15	NS
SNB	−2.19	1.51	−1.32	1.99	0.13	NS
ANB	2.58	1.80	2.17	2.41	0.95	NS
SNL–ML	2.76	1.39	−0.25	2.90	0.007	*
SNL–OL	−1.73	3.90	−1.04	3.33	0.33	NS
SNL–NL	0.72	2.72	−0.66	2.15	0.87	NS
Is–SNL	−0.19	7.35	−4.42	5.67	0.04	*
Ii–ML	−4.33	7.68	−2.23	3.84	0.09	NS

*NS* not significantly different

*Significantly different at *p* < 0.05

#### Sagittal and angular differences

**Fig. 5:**
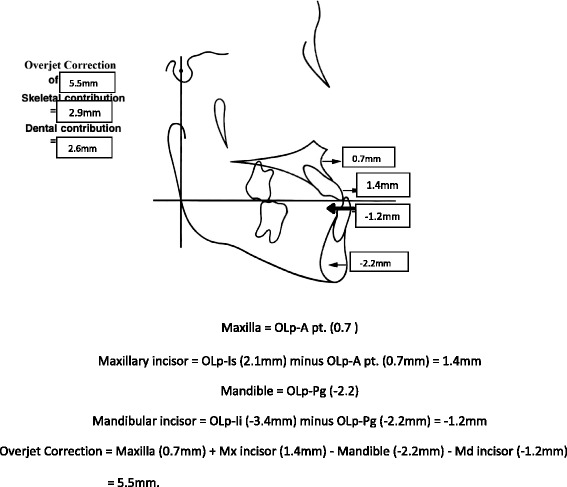
Skeletal and dental contributions to overjet correction for the tooth-borne protraction facemask group

**Fig. 6:**
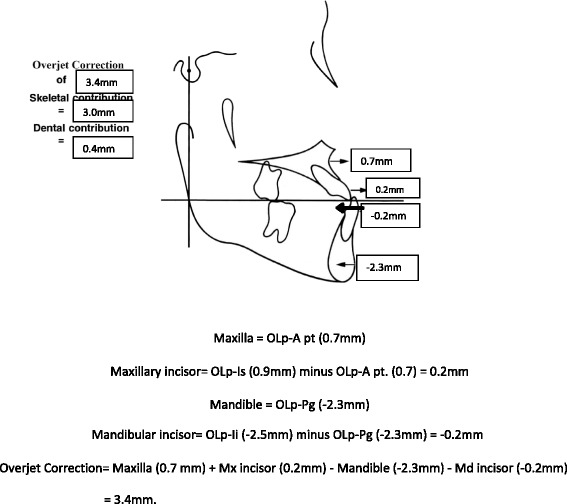
Skeletal and dental contributions to overjet correction for the bone-anchored protraction facemask group

**Fig. 7:**
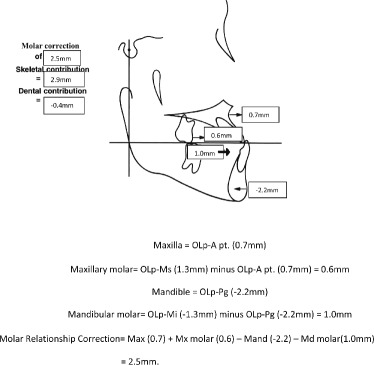
Skeletal and dental contributions to molar relationship correction for the tooth-borne protraction facemask group

**Fig. 8:**
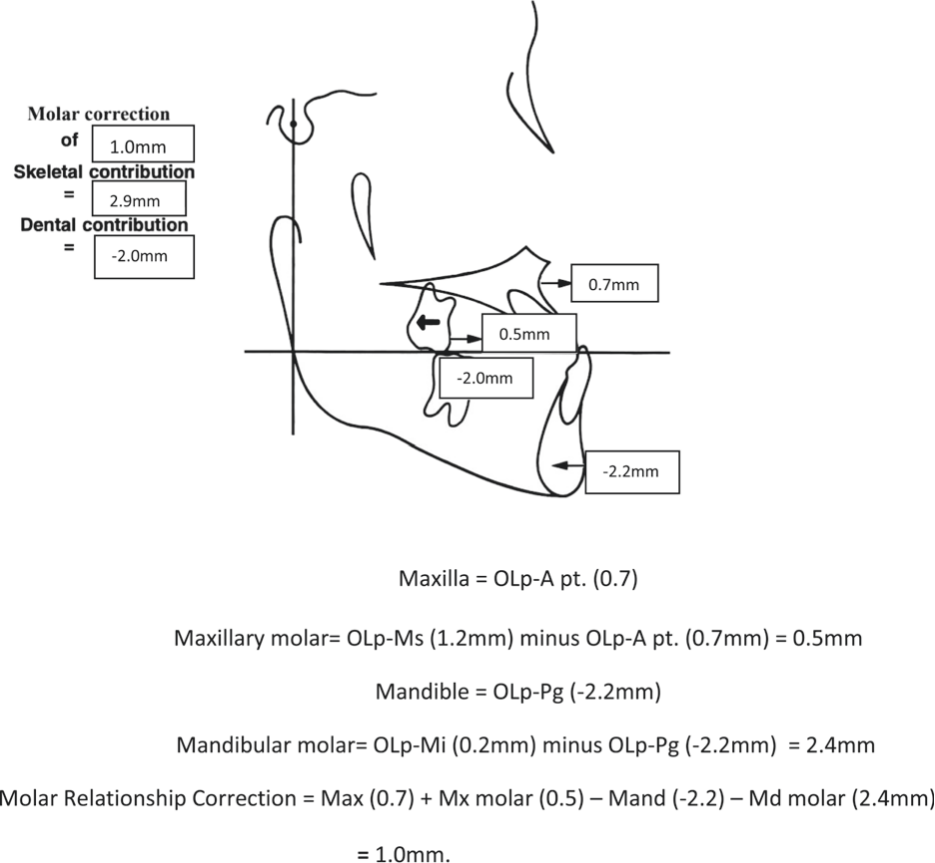
Skeletal and dental contributions to molar relationship correction for the bone-anchored protraction facemask group

Significant differences between the tooth-borne and bone-anchored protraction facemask groups were found in three of the 10 sagittal and angular variables. Figures 5, 6, 7, and 8 summarize the skeletal and dental contributions to the overjet and molar correction from treatment.

For both treatment groups, all subjects were overcorrected to a class I or class II molar relationship. Significant and greater change in overjet was found in the tooth-borne group (5.5 mm) compared to the bone-anchored group (3.4 mm, *p* < .001). This was contributed by similar forward movement of the maxilla (OLp-A pt., 0.7 mm) and backward movement of the mandible (OLp-Pg, 2.2 mm) in both groups, but greater forward movement of the maxillary incisors was found in the tooth-borne group (OLp-Is 2.12 vs. 0.87 mm, *p* < .05).

Significant and greater change in molar relationship was found in the tooth-borne group (2.7 mm) compared to the bone-anchored group (1.1 mm, *p* < .05). This was contributed by similar forward movement of the maxilla and backward movement of the mandible in both groups, but greater differential movement of the maxillary and mandibular molars was found in the tooth-borne protraction facemask group. The mean forward movement of maxillary molars was similar for the tooth-borne (0.6 mm) and bone-anchored protraction facemask groups (0.5 mm).

The anteroposterior jaw relationship was improved in both the tooth-borne group (Wits 2.19 mm, ANB 2.58°) and the bone-anchored group (Wits 2.31 mm, ANB 2.17°).

#### Vertical and angular differences

Significant differences between the tooth-borne and bone-anchored protraction facemask groups were found in five of the 14 vertical and angular variables. Significantly greater downward movement of the maxilla was found in the tooth-borne (OLparallel–A pt. 1.2 mm) compared to the bone-anchored protraction facemask group (−0.4 mm, *p* < .005). The mandibular plane angle was found to open significantly more in the tooth-borne group (SNL–ML 2.76°) compared to the bone-anchored protraction facemask group (−0.25°, 0.23°, *p* < .05). Significantly greater downward movement and retroclination of the maxillary incisors was found in the bone-anchored group (Is-NL 1.34 mm, Is-SNL −4.42°) compared to the tooth-borne group (−0.55 mm, −0.19°, *p* < .05).

## Discussion

Maxillary protraction using both the tooth-borne RPE and RPE reinforced by two mini-implants were able to correct the anterior crossbite and improve the molar relationship in around 6 months. This was contributed by a forward and downward movement of the maxilla, clockwise rotation of the mandible, proclination of the maxillary incisors, and retroclination of the mandibular incisors. The maxilla articulates with nine other bones of the craniofacial complex: frontal, nasal, lacrimal, ethmoid, palatine, vomer, zygoma, inferior nasal concha, opposite maxilla, and occasionally, sphenoid. Palatal expansion may “disarticulate” the maxilla and initiate cellular response in the sutures, allowing a more positive reaction to protraction forces [12, 13, 37]. In this study, the maxilla moved forward an average of 1.5 mm, or 0.7 mm after subtracting changes due to growth, with both the tooth-borne and bone-anchored protraction facemask treatment. This is in line with those reported by Nanda [38], Ishii [39], Merwin [40], and Turley [12]. However, Cevidanes et al. reported larger maxillary advancement with their bone-anchored maxillary protraction (BAMP) compared to conventional RPE [29]. This is probably related to the full-time wear of class III elastics compared to smaller number of hours with the facemask. The maxillary incisors were found to move forward more in the tooth-borne compared to the bone-anchored protraction facemask group, resulting in greater increase in the overjet in the latter group. This may be due to the anchorage provided by the two mini-implants. The maxillary molars were found to move forward an average of 0.6 mm in the bone-anchored groups despite the anchorage provided by the two mini-implants. This is in line with those reported by Wilmes et al. [32, 33] with the Hybrid Hyrax appliance and other investigators that used bone-anchored devices for maxillary protraction [19–21, 27–30]. This small amount of mesial molar movement is probably due to wire bending rather than movement of the mini-implants.

For vertical changes, the mandible was found to move backward in both the tooth-borne and bone-anchored groups (SNB −2.2° and −1.3°, respectively). This, together with the forward movement of the maxilla, contributed to the improvement in the Wits appraisal and ANB changes in both groups. However, the downward movement of the maxilla in the latter group was significantly less. The incorporation of two mini-implants helps in minimizing the downward movement of the maxilla and consequently the clockwise rotation of the mandible in the bone-anchored group (SNL–ML 0.7° for the bone-anchored group vs. SNL–ML 2.9° for the tooth-borne group, *p* < .05). This is also in line with those reported by investigators using bone-anchored devices for maxillary protraction [19–21, 27–33]. In addition, there is more downward movement of the maxillary incisors in the bone-anchored group compared to the tooth-borne group that helps to maintain the overbite in the bone-anchored group.

There are several limitations to the design of this study. This is a retrospective study and the patients were treated by two different operators, one for the tooth-borne patients and one for the bone-borne patients. The radiographs of the two treatment groups were taken from different lateral cephalometric machines but they were adjusted for magnification. The control radiographs were selected from class III patients who were under growth observation, and efforts were made to match the age, sex, and craniofacial morphology as closely as possible. Finally, the results from this study are limited to a short-term observation period immediately after active treatment; long-term studies are needed for the appraisal of the stability of protraction with the Hybrid Hyrax appliance and compared to the long-term results of conventional RPE and facemask treatment [14].

## Conclusions

The addition of two mini-implants in the Hybrid Hyrax RPE minimized the side effects encounter by tooth-borne RPE for maxillary protraction such as excessive forward movement of the maxillary molars and incisors, downward movement of the maxilla, and clockwise rotation of the mandible. This appliance may serve as an alternative treatment appliance for correcting class III patients with a hyperdivergent growth pattern.
